# Advanced Care Planning in Parkinson's Disease: In-depth Interviews With Patients on Experiences and Needs

**DOI:** 10.3389/fneur.2021.683094

**Published:** 2021-07-28

**Authors:** Elisabeth Kurpershoek, Marij A. Hillen, Niki M. Medendorp, Rob M. A. de Bie, Marianne de Visser, Joke M. Dijk

**Affiliations:** ^1^Department of Neurology, Amsterdam Neuroscience, Amsterdam UMC, University of Amsterdam, Amsterdam, Netherlands; ^2^Department of Medical Psychology, Amsterdam Public Health, Amsterdam UMC, University of Amsterdam, Amsterdam, Netherlands

**Keywords:** Parkinsion's disease, advanced care planning, information preferences, physician-patient communication, qualitative analysis

## Abstract

**Introduction:** Advance care planning (ACP) is an iterative process of discussing the needs, wishes, and preferences of patients regarding disease-specific and end-of-life issues. There is ample evidence that ACP improves the quality of life and promotes the autonomy of patients with cancer and motor neuron disease who have a high disease burden and shortened life expectancy. In Parkinson's disease (PD) though, knowledge about the experiences and preferences of patients regarding ACP is scarce, despite the major disease burden associated with PD.

**Aim:** This study aims to explore the experiences, needs, and preferences of PD patients regarding the content and timing of ACP.

**Methods:** In-depth interviews were conducted with a purposively selected sample of patients diagnosed with PD. Using a semi-structured topic list, the participants were asked about their prospects for a future living with PD and with whom they wanted to discuss this. Qualitative analysis was performed in parallel with data collection using a data-driven constant comparative approach. The transcribed interviews were coded and analyzed by two researchers using MAXQDA software.

**Results:** Of all 20 patients (13 males; age 47–82; disease duration 1–27 years), most expressed a wish to talk about ACP with a healthcare provider, enabling them to anticipate the uncertain future. The majority of patients preferred their healthcare provider to initiate the discussion on ACP, preferably at an early stage of the disease. Nearly all patients expressed the wish to receive more information regarding the long-term impact of PD, although, the preferred timing varied between patients. They also perceived that their neurologist was primarily focused on medication and had little time to address their need for a more holistic approach toward living with PD.

**Conclusion:** Our results suggest that PD patients are in need of discussing ACP with their healthcare provider (HCP), even in the early stages of the disease. In addition, PD patients perceive a lack of information on their disease course and miss guidance on available supportive care. We recommend HCPs to inquire the information requirements and preferences of patients regarding ACP regularly, starting soon after diagnosis.

## Introduction

Parkinson's disease (PD) is a neurodegenerative disorder with both motor symptoms, such as bradykinesia, rigidity, and tremor, and non-motor symptoms, including autonomic dysfunction and psychiatric manifestations (1, 2). It is difficult to predict how PD will develop in individual patients. As the disease progresses, the motor symptoms generally increase in severity, and patients may additionally experience levodopa-induced dyskinesia, gait impairments, falls, dysphagia, and dysarthria. Moreover, they may develop psychiatric symptoms and/or cognitive impairment. Eventually, the majority of patients will develop dementia (3). Thus, PD patients experience progressive impairments in their day-to-day activities and become increasingly reliant on their caregivers. Eventually, ~40% will be living in a nursing home (4, 5).

There is growing evidence that *early* integration of palliative care in chronic progressive neurologic disorders improves the quality of life of patients and their significant others (6, 7). Advance care planning (ACP) is an element of palliative care in which the needs, wishes, and preferences of patients regarding disease-specific and end-of-life issues are discussed in an iterative process.

The introduction of advance care planning is possible alongside curative therapies and at any time during the disease course, sometimes even directly after communication of the diagnosis (8, 9). However, in PD, instead of being integrated early, research suggests that, in current practice, ACP generally is not initiated before the progression of symptoms, cognitive decline, or the terminal phase of PD (10).

In amyotrophic lateral sclerosis (ALS) and in non-neurological diseases, mostly cancer, ACP was found to be associated with a higher quality of life, fewer hospitalizations, more compliance with the preferred place of death, and less stress, anxiety, and depression (11, 12). Since PD, like ALS, is a chronic progressive disease associated with substantial morbidity, one can argue that ACP may serve the same purpose in PD patients (13). The timely onset of ACP may be crucial for PD patients as their capacity to express their wishes regarding care may decline due to motor or cognitive impairments (14). Preliminary evidence suggests that half of PD patients prefer to discuss advance directives early in the disease course, whereas, 20% prefer to wait until the disease progresses (15). In practice, palliative care, including ACP, currently seems to be underutilized in PD patients, and neurologists were found to postpone conversations on initiating, withholding, and/or withdrawing treatment in PD until there is significant physical or cognitive decline (16, 17). There is sparse knowledge on the content and optimal timing of ACP in PD (18).

In this study, we set out to obtain insight into the experiences, needs, and preferences of patients with PD regarding ACP at different stages of the disease.

## Methods

A qualitative design using in-depth semi-structured interviews was employed. The study was performed according to the Consolidated Criteria for Reporting Qualitative Research (see Data Sheet 2) (19). The institutional Medical Ethics Review Board waived the need for ethico-legal adjudication. All participants gave written informed consent for participation in the study.

### Setting

The study was performed at a tertiary referral center for PD and for deep brain stimulation (DBS) treatment, a surgical intervention for advanced PD. Many patients were initially treated elsewhere and were referred for DBS treatment. Patients with DBS often maintain a treatment relationship with the referring neurologist. The PD patients are usually treated by various healthcare providers (HCPs), including neurologists specializing in movement disorders, general neurologists, specialist nurses (regarding DBS and PD treatment), and neurology residents.

### Recruitment of Participants

All HCPs at the study site who were involved in the care for PD patients were requested to invite patients treated at the outpatient clinic to participate. The patients were informed that the study focused on communication between patients and their treating HCP about treatment options and the preferences of patients regarding their (future) healthcare. The patients were eligible if they had sufficient command of the Dutch language, had been diagnosed with PD at least 1 year ago, and had no known cognitive impairment. We purposively sampled patients to obtain a broad variation regarding disease duration, age, gender, and disease stage. The eligible patients were contacted by the first author (EK) to further inform them and their significant other about the study. If a patient provided initial oral consent, an appointment was made for the interview.

### Data Collection and Analysis

Semi-structured interviews were conducted by the first author (EK), a female graduate medical student trained in qualitative interviewing techniques. The interviews took place at the preferred time and location of the patient, and significant others were allowed to participate in the interview depending on the preferences of the patients. Before the interview started, possible cognitive impairment was assessed by EK as a background characteristic, using the Montreal Cognitive Assessment (MoCA) (20). Other patient characteristics were assessed using a brief self-reported questionnaire, and disease stage was scored according to the Hoehn and Yahr (H&Y) scale, based on data in the electronic patient file (21). The interviews were audio-recorded, subsequently typed-out verbatim, and anonymized by the first author (EK). The transcripts were not returned to the participants.

An interview guide was created in advance by EK and four experienced researchers [two neurologists (JD and MdV) and two psychological researchers experienced with qualitative research (NM and MH)], and it was pilot-tested on two patients prior to the start of the study. The interview guide focused on (1) experiences with advance care planning and (2) preferences in discussing and documenting ACP (for the full topic list, see Data Sheet 1). To ensure data-driven analysis, the constant comparative method was employed (22, 23). Analysis was performed in parallel with data collection by three researchers (EK, NM, and MH), using MAXQDA software, version 12 (VERBI software). The interview guide was continuously refined based on the initial analysis. The first five interviews were all independently coded by three researchers (EK, NM, and MH) and subsequently compared and discussed. The subsequent interviews were coded by EK, three of which were double-coded by NM and compared to enhance triangulation. After open coding of all transcripts, the codes were ranked into subcategories that were merged into mutually exclusive categories. Data collection was terminated when saturation was reached, i.e., when three subsequent interviews did not yield any substantial new information (24). Eventually, data were clustered across interviews by EK, NM, MH, JD, and MdV to derive common themes. The patient advocates were requested to provide feedback on the findings in the common themes, which led to some amendments. The original Dutch quotes were translated by a native English speaker.

## Results

Twenty-seven patients were contacted by EK, five of whom declined participation. The reasons for declining could not be assessed. Two patients were excluded after the interview because their proficiency in the Dutch language turned out to be insufficient. Twenty patients (13 males and seven females) were included in the study (see Table 1 for demographics and clinical characteristics). The median age was 63 years (range, 47–82), and the median disease duration was 9 years (range, 1–27). One patient mentioned having had appointments exclusively with a neurologist, while the rest mentioned having been treated by different types of HCPs: neurologists, neurology residents, and specialist nurses. Most patients had moderately severe motor symptoms according to the Hoehn and Yahr scale. Five patients were still working. None of the patients had a severe cognitive impairment, albeit 25% had a MoCA score indicative of mild cognitive impairment (score between 21 and 25). Eleven interviews were held in the presence of the informal caregivers of the patients, who actively took part in the conversation. All interviews were held at the homes of the patients and lasted between 45 and 120 min. Data saturation was reached after 17 interviews.

**Table 1:** Characteristics of 20 interviewees.

	***n***	**(%)**
**Gender**		
Male	13	(65)
Female	7	(35)
**Age (years)**		
40–49	1	(5)
50–59	7	(35)
60–69	8	(40)
70–79	2	(10)
>80	2	(10)
**Education**		
Low (none or primary education)	2	(10)
Middle [(basic) vocational training]	9	(45)
High (research University and University of applied sciences)	9	(45)
**Time since Parkinson's disease diagnosis (years)**		
1–4	5	(25)
5–9	7	(35)
10–14	5	(25)
15–19	2	(10)
25–29	1	(5)
**Cognitive impairment (Montreal cognitive assessment)**		
21–25 points (mild cognitive impairment)	5	(25)
26–30 points (no cognitive impairment)	15	(75)
**Disease stage (Hoehn and Yahr scale** ^**a**^ **)**		
Stage 1	2	(10)
Stage 2	4	(20)
Stage 3	12	(60)
Stage 4	2	(10)

a *Hoehn and Yahr scale: stage 1, unilateral involvement only, usually with minimal or no functional disability; stage 2, bilateral or midline involvement without impairment of balance; stage 3, bilateral disease—mild to moderate disability with impaired postural reflexes, physically independent; stage 4, severely disabling disease, still able to walk or stand unassisted; stage 5, confinement to bed or wheelchair unless aided*.

### Interview Results

Two major themes emerged from the interviews (Table 2): first, communication with various healthcare professionals about the diagnosis and advance care planning and, second, communication about the uncertainty of the future disease burden.

**Table 2:** Themes that emerged from the patient interviews (individual patients raised different topics in the interviews; the major themes are summarized below).

**Communication with healthcare provider (HCP) about diagnosis and advance care planning (ACP)**
Information provision is considered suboptimal and may be improved by, e.g., a two-tiered diagnostic appointment.
Patients value a healthcare provider with a holistic and empathic approach and who has sufficient knowledge of Parkinson's disease and enough time.
Most patients wish to discuss ACP with their HCP—most with the specialist nurse, some with the neurologist.
Many patients prefer their HCP to explore their willingness to start an ACP conversation.
The preferred timing of the first ACP conversation differs widely.
**Communication with HCP about the uncertainty of the disease burden**
Patients are concerned about their uncertain future disease burden, they fear becoming demented and losing their (physical) independency.
Many patients find it important to anticipate on the future, mainly regarding practical issues.
The fear patients express of becoming increasingly dependent, makes them consider hastened death.

### Communication About Diagnosis and Advance Care Planning

Most patients reported that they did not receive enough information on the consequences of the PD diagnosis. The bad news elicited many questions.

*(They told me) nothing, absolutely nothing. I remember feeling outraged. I went home thinking: what's next? When will I die? That was my first reaction. I understand you can't cover every single detail during the first consult. You don't listen as well after hearing bad news. But to send someone home without any written information, no booklet or folder … something to read after you've recovered from the initial shock. I thought that was terrible*.

Respondent 11, female, 56 years old, H&Y stage 3, 14 years since diagnosis

Only a few patients reported having received adequate information about the diagnosis and its consequences, mostly during a separate follow-up appointment in which the neurologist took ample time to discuss all aspects of PD. The patients who reported having had such a follow-up appointment were highly satisfied with its timing, ~4 to 6 weeks after diagnosis.

*The neurologist said to me: I can provide lots of information now. But I'm sure you wish to clear your mind first. So, we made another appointment, 4 weeks later. This appointment took over an hour. He explained everything: the consequences, medication, different perspectives, the Parkinson Association, what my wife could expect. We discussed what kind of outlook to have on life with PD, and how there was more to life than being a PD patient. We covered all sorts of topics. It was very pleasant to divide this over two moments. When they first tell you: “you have PD”, your world falls apart. But you don't know to what extent it's falling apart. You're filled with emotions: you're afraid of what you don't know; you're angry. It's good to calm down and do some research before going back to the neurologist (for the second appointment)*.

Respondent 29, male, 54 years old, H&Y stage 3, 12 years since diagnosis

Various types of healthcare professionals were involved in the care of patients, e.g., neurologists, specialist nurses, and general practitioners. A few patients described that their neurologist took time by scheduling a second appointment for a more comprehensive explanation about the diagnosis and associated consequences. However, most patients described the role of the neurologist as that of a technical specialist, with little time available for their patients and only responsible for the diagnosis and PD medication.

*These conversations with the neurologist are only about the medication. Not about how it is going at home. I had just divorced and I had my two sons, only 11 years old, living with me. Nobody bothered to ask how that was going, and how to anticipate the moment when I would not be able to take care of them any longer*.

Respondent 12, male, 60 years old, H&Y stage 3, 16 years since diagnosis

They expressed the wish to receive more holistic, empathetic care from the neurologist:

*(The neurologist provides) a diagnosis and medication. These things are really important. However, in my opinion, something is missing. I mean, like, empathy, or compassion. He shouldn't wash his hands off everything and just refer you to the specialist nurse. A good neurologist understands what it's like to live with PD*.

Respondent 29, male, 54 years old, H&Y stage 3, 12 years since diagnosis

The specialist nurse was described as caring and empathetic and was reported by the patients to spend more time compared to the consultant neurologist. About half of the patients indicated feeling more comfortable discussing the impact of PD on their lives with their specialist nurse.

*[The role of the specialist nurse is] further guidance of and support for patients, in what they deal with every day. Also, providing the proper referrals and checking the patient's own environment. How are things at home? Are your relationships suffering? Do you have a job or hobbies that you enjoy doing?*

Respondent 29, male, 54 years old, H&Y stage 3, 12 years since diagnosis

Some patients perceived that HCPs involved in their care, such as their general practitioner, had little knowledge regarding PD, e.g., about when to refer patients for specialized paramedical care.

*Well, I have noticed that most healthcare professionals, my GP included, simply have too little knowledge about PD to provide useful information. Both the neurologist and the specialist nurse have no clue where to find this specialized care. That needs improvement. That way, patients don't have to figure everything out by themselves*.

Respondent 28, female, 58 years old, H&Y stage 3, 7 years since diagnosis

Nearly all patients expressed a wish to talk about ACP with their HCPs, enabling them to anticipate the future.

*Sometimes I find myself thinking: tell me more about the consequences of PD. At the physical therapist and on television I see patients with PD who are much more disabled than I am. And the neurologist only asks me how it is going right now. He never tells me what to expect regarding the development of PD*.

Respondent 3, female, 82 years old, H&Y stage 3, 3 years since diagnosis

A few explicitly preferred to discuss ACP with the neurologist.

*If you're asking me what our next step should be, I would like to discuss my future with my neurologist. I think that would make sense*.

Respondent 11, female, 56 years old, H&Y stage 3, 14 years since diagnosis

A larger proportion of patients expressed a wish to have such conversations with the specialist nurse.

*The neurologist isn't really involved. You only visit him twice a year. He's almost a stranger to me. I would have liked to speak to a specialist nurse who could tell you everything there is to know about all the different regulations and options available for Parkinson's patients in Parkinson's care*.

Respondent 2, male, 64 years old, H&Y stage 3, 7 years since diagnosis

Only a few patients thought ACP was not useful.

‘*It's sort of a self-fulfilling prophecy. You know you'll eventually become stiff, so you feel stiff already. (…) It's hard, but I feel that the less pre-occupied I am with the disease, the less I feel its limitations on me. (…) I don't like to brood over this. Sometimes I do, of course. And when I do, I become unpleasant and angry*.

Respondent 18, male, 58 years old, H&Y stage 3, 11 years since diagnosis

Most patients had discussed issues relating to ACP with their loved ones, whereas, only a few had actually discussed ACP with their HCP.

*What if I develop dementia and I am not aware of it anymore. Well, I told my son and husband that I do not want to be left neglected. If I no longer look well-cared for, then I want to be euthanized*.

Respondent 3, female, 82 years old, H&Y stage 3, 3 years since diagnosis

Most patients reported that they found it difficult to start a conversation about ACP with their HCP themselves. Instead they would prefer the HCP to initiate this conversation. They emphasized that the HCP should be careful in doing so, exploring whether the patient is willing to discuss these issues.

*I think it's fine if the neurologist or the specialist nurse discusses the future. Some patients might back down from this conversation because they're not ready to discuss it yet. Perhaps they (HCPs) should ask how the patient is feeling at that moment. Then, they can continue by asking what should be arranged for you when things get worse*.

Respondent 11, female, 56 years old, H&Y stage 3, 14 years since diagnosis

The patients varied in their preferences regarding the ideal timing of ACP conversations. Some reported that they did not want to discuss generic, disease-specific, and end-of-life issues until their PD symptoms worsened.

*I'll bring it up when it's necessary. That will give me plenty of time still*.

Respondent 24, female, 69 years old, H&Y stage 4, 27 years since diagnosis

Contrarily, some patients preferred to hear about the prognosis and therapeutic or supportive options as soon as possible after the diagnosis. Most patients reported that they would not be bothered if an HCP would attempt to initiate a discussion about ACP early in the disease course.

*As soon as possible. It's tough, but at least it's clear. That way, you can arrange everything while you're still thinking clearly. You can't leave it all in the hands of your children. You have to take responsibility*.

Respondent 16, male, 57 years old, H&Y stage 3, 16 years since diagnosis

### Communication About the Uncertainty of the Future Disease Burden

The second major theme that emerged from the interviews was the uncertainty of the patients about their future disease burden. The patients reported several concerns about the future that they had not been able to discuss with their HCP. They expressed concerns about ending up in a wheelchair or not being able to take care of themselves anymore and thus becoming a burden for their loved ones or having to live in a nursing home. Almost all patients were afraid to become demented.

*Yeah, I find myself wondering: what will become of me? What if I will develop dementia … My daughter volunteers at a nursing home for patients with dementia every Sunday. Should I write a euthanasia codicil? Will I remain kind, or will I become a really nasty patient? If that happens, I want it written down somewhere that I do not wish to continue to live*.

Respondent 21, female, 56 years old, H&Y stage 2, 1 year since diagnosis

The patients suggested that these important uncertainties about their future disease burden should be addressed in ACP conversations. The patients also reported that ACP conversations should pertain not only to their symptoms but also to the impact of these symptoms on their daily lives. They expressed the wish to get support for activities of daily living, access to devices, or nursing care for personal hygiene. Other aspects which the patients reported that they wanted to address in ACP conversations were resuscitation, hastened death, and nursing care.

*The physical care, absolutely. What does it entail? Can your spouse manage? If she can't, you have to make other arrangements. If it were up to me, I would postpone that as much as possible. I would also want euthanasia performed at home, not in a hospital. If the neurologist is clear, you know what to expect of him. They should also write everything down so you can get back to certain topics*.

Respondent 16, male, 57 years old, H&Y stage 3, 16 years since diagnosis

Many patients who emphasized the importance of anticipating on their future focused on practical issues.

*Well, practical items like beds, walkers, toilets, stair lifts. I'm trying to get ahead by purchasing these items already before I'm fully dependent on them*.

Respondent 28, female, 58 years old, Hoehn and Yahr stage 3, 7 years since diagnosis

The patients in our cohort with a relatively short disease duration (i.e., <5 years) and who experienced low symptom burden seemed not to differ in their experiences, needs, and preferences regarding ACP and the uncertainty about future disease burden compared to patients with a more advanced disease.

## Discussion

We aimed to explore the experiences, needs, and preferences of PD patients regarding the content and timing of ACP. The findings of our study suggest that nearly all patients desired to discuss ACP with their HCP, even those who had been recently diagnosed and as yet had experienced a relatively low symptom burden. Moreover, the patients perceived a lack of information on their disease course and felt a need for more guidance in finding available supportive care.

Even though, most patients had a desire to discuss ACP with their HCP, only one patient in our sample actually had had such a conversation. The patients generally preferred their HCP to initiate an ACP conversation as they found it difficult to start an ACP conversation themselves. Nevertheless, our group previously showed that neurologists usually do not discuss ACP before the terminal stages of PD. The longstanding relationship between patients and their neurologist in which the focus is on optimizing medical treatment to suppress symptoms may be a barrier for the neurologist to start an ACP conversation (16). This may partially explain why this conversation had not taken place with most patients.

The preferences of the patients regarding the timing of ACP ranged from right after diagnosis to when the disease has progressed. Even those who preferred to discuss ACP later in the disease course still reported that they would not be bothered if the physician would initiate a discussion about ACP at an early stage. These results suggest a discrepancy between the wishes of patients to discuss ACP with their healthcare providers at an early disease stage and their actual experiences.

In contrast to PD, in high-grade glioma and ALS, ACP is initiated directly at diagnosis because of the reduced life expectancy and imminent cognitive impairment, especially in glioma (16). It may well be that the neurologists underestimate the urgency to discuss ACP early in the disease course or to discuss it at all since PD generally initially is well-treatable, and most patients have many years to live after diagnosis. Moreover, the unpredictability of the course of PD possibly contributes to delaying these discussions (20).

That PD patients experience the need for a timely discussion of future care is supported by other studies (13, 15, 25). Evidence regarding the optimal timing of ACP is still scarce. A recently developed tool aimed at timely identifying palliative care needs in PD patients by HCPs may be of practical use (26).

The preferred content of ACP conversations included mostly practical topics, such as support for activities of daily living, access to devices, or home healthcare. Additionally, the patients also expressed their wish to discuss resuscitation and hastened death. Of note is that none of the patients had articulated a wish to be informed about the salient features of advanced PD, such as balance problems, swallowing difficulties, urinary symptoms, or aspiration pneumonia, in an ACP conversation. The patients may not have been sufficiently informed about these issues and therefore had not brought them up. ACP can only be effective if the patient is well-informed not only about the diagnosis and its implications but also about the prognosis (27).

Many patients in our sample indeed felt that they had not received enough information and guidance regarding the course of PD. Since patients with a relatively short disease duration retrospectively reported a lack of provided information as well, one might argue that this need for information is already present shortly after diagnosis. This perceived lack of information by the patient might be multi-causal. Firstly, the information provided may indeed have been insufficient. Additionally, the information may have been provided but forgotten by the patients. Previous research demonstrates the inability of patients to effectively take up additional information directly after receiving a life-altering diagnosis due to the associated stress (28). Moreover, if the information provided to patients throughout their disease course does not match their information needs at that particular moment, they may fail to absorb it. Finally, the reports of the patients during the interviews may have been biased: information that was provided years ago might be inaccurately recalled due to the elapsed time or cognitive decline. Prospective studies are required to further investigate the causes of the perceived lack of information.

Our results do substantiate earlier findings among PD patients and their informal caregivers regarding information preferences—that many patients and their caregivers have a strong need for iterative, tailored information already shortly after the diagnosis (29–31). Additionally, they align with previous findings among patients with chronic progressive neurological diseases, showing that patients highly value participation in the decision-making about treatment and care, which is only possible if the patient is informed about possible disease progression (8, 13, 15, 32).

Our study has several strengths. Our sample included a wide variation in disease duration, disease severity, and age, which contributes to the validity of our results. The participating patients were also treated by various types of HCPs (e.g., specialist nurse, resident, neurologist, and/or specialist neurologist in movement disorders). The interviews took place at the homes of the patients and were conducted by an independent interviewer, which may have encouraged the patients to openly and critically talk about their experiences and preferences. A thorough analysis was ensured by involving a multidisciplinary team, including two researchers experienced in qualitative research methodology. Several limitations have to be mentioned as well. First, some degree of selection bias may have occurred since the potential candidates for our study were selected by their treating HCP. This might have led to the inclusion of patients with a tendency to express their opinions more explicitly. Five of the 27 eligible patients declined participation, but we could not ask them for their reasons to decline. Besides this, while standard qualitative methods were used for this study, some interviewer bias may have influenced the interview content, selection of themes, and/or presentation of results, yet we minimized bias by using investigator triangulation with a multidisciplinary analytical team (33, 34).

In addition, the patients mentioned having received treatment from different types of HCPs. Most patients had experience with care from one or more neurologists and specialist nurses. Even though, the patients did not explicitly mention this, a substantial proportion likely received treatment from a neurology resident as well since the patients were treated in a teaching hospital.

Additionally, that all patients were included in one medical center may impair the generalizability of the results. Since this was a tertiary referral center for Parkinson's disease, about half of these patients had previously been or were simultaneously treated elsewhere, still ensuring variability in experiences. Finally, not all results can be readily extrapolated internationally as, in the Netherlands, end-of-life considerations, including hastened death, are relatively openly discussed. Conversely, since the results of our study resemble those from earlier publications from the UK and USA regarding the readiness to openly discuss end-of-life issues early on, they seem generalizable at least to western countries with a high socioeconomic status (13, 15, 25).

Based on the results of this study, reporting the experiences and preferences of patients, we first recommend the HCPs to explore the preferences of patients regarding ACP regularly, starting early in the disease trajectory. Second, as a well-informed patient is a prerequisite for an ACP conversation, information provision to patients should be optimized before ACP can be properly implemented in the standard care of PD. For example, one might consider informing PD patients about the diagnosis and consequences of the disease in a two-tiered appointment similar to the process in oncology and ALS since this was shown to facilitate information uptake by the patients in the latter patient groups (8). Communication skills training for HCPs may be crucial to optimize these conversations (10). Additionally, by regularly actively inquiring the need for information by the HCPs regarding prespecified topics, tailored information in both oral and written form can be supplied to PD patients. This may facilitate the information uptake, taking the prolonged disease duration with changing symptoms over time and potential cognitive decline into account.

Finally, to optimize communication about ACP, future research is necessary regarding the following: (1) the communication strategy of neurologists on breaking the bad news of a PD diagnosis and information provision regarding the associated consequences of this diagnosis during the disease course, (2) how the patients and their significant others experience these conversations, and (3) whether the abovementioned recommendations lead to better informed patients.

## Conclusion

We conclude that PD patients often feel insufficiently informed about their diagnosis, possible future disease evolvement, and future disease burden. Most PD patients wish to discuss ACP with their HCP. The patients varied in their preferences regarding the ideal timing of ACP conversations, yet a substantial part wanted to start shortly after the diagnosis. The interviewed patients expressed the wish that the HCP takes the initiative to start such a conversation. Though future research is needed before ACP can be adequately and efficiently applied in standard care in PD, some recommendations can be made. It seems important to proactively, timely, and iteratively inquire about the needs of the patients for information on the different aspects of the disease. Only then can tailored educational materials be provided at the right time. Finally, it is advised that the HCP regularly verifies the need of the patients to discuss ACP.

## Data Availability Statement

The datasets presented in this article are not readily available due to the sensitive nature of the full interview transcripts. Requests to access the datasets should be directed to e.kurpershoek@amsterdamumc.nl.

## Ethics Statement

Ethical review and approval was not required for the study on human participants in accordance with the local legislation and institutional requirements. The patients/participants provided their written informed consent to participate in this study. Written informed consent was obtained from the individual(s) for the publication of any potentially identifiable images or data included in this article.

## Author Contributions

EK: conceptualization, methodology, formal analysis, investigation, data curation, project administrations, writing-original draft, and writing-review and editing. MH: conceptualization, methodology, formal analysis, data curation, writing-review and editing, supervision, and providing resources. NM: conceptualization, methodology, formal analysis, data curation, and writing-review and editing. RB: writing-review and editing, supervision, and providing resources. MV: conceptualization, methodology, formal analysis, supervision, writing-review and editing, and providing resources. JD: conceptualization, methodology, formal analysis, supervision, writing–original draft, writing-review and editing, and providing resources. All authors contributed to the article and approved the submitted version.

## Conflict of Interest

The authors declare that the research was conducted in the absence of any commercial or financial relationships that could be construed as a potential conflict of interest.

## Publisher's Note

All claims expressed in this article are solely those of the authors and do not necessarily represent those of their affiliated organizations, or those of the publisher, the editors and the reviewers. Any product that may be evaluated in this article, or claim that may be made by its manufacturer, is not guaranteed or endorsed by the publisher.
