# Supplementary material for: Advanced Care Planning in Parkinson's Disease: In-depth Interviews With Patients on Experiences and Needs
Source: Front Neurol. 2021 Jul 28;12:683094. doi: 10.3389/fneur.2021.683094 (PMC8355553; doi:10.3389/fneur.2021.683094)
Supplement: Supplementary file 1 [file Data_Sheet_1.pdf]

Appendix 1 – Topic list of the interview

The questions marked with a * were asked to all patients. The sub-questions were used for further exploration when necessary.

Questions regarding experiences with advance care planning

What are your thoughts about your future, living with PD?*

- Are you uncertain/afraid of what might come? What is it that makes you insecure/afraid?
- How do you feel about the future if the disease advances?*
- What determines your quality of life? When would you describe your quality of life as poor?

What has your neurologist told you about what to expect in the coming years?*

- Did you discuss the possible decisions you might be confronted with regarding your Parkinson care in the coming years?*
    - Who took the initiative for this conversation?*
    - Which issues were discussed?*
    - How do you feel about this conversation?*
        - What elements did you miss? What elements would you rather not have heard? How did you feel during this conversation? How much space was there to discuss your needs and preferences?
    - Which of your significant others attended this conversation? How did you feel about that?
    - Was something written down after the conversation? And if so, what?*
        - Did the neurologist take notes from the conversation, to document your values and preferences in your patient file?

How would you describe the relationship between you and your neurologist?*

- How did this prior relationship influence your conversations about the future?
- How did the neurologist's communication during this conversation contribute to your trust in him/her?

How do your family and friends influence your preferences regarding potential future decisions in your Parkinson care?*

Are there any other healthcare professionals involved in your care for Parkinson's disease?*

- GP/geriatrician/rehabilitation physician/ speech therapist / physiotherapist etc.?

Questions regarding preferences in discussing and documenting advanced care planning

Which healthcare professional would you prefer to discuss with you potential decisions about your future care?*

- Why and why not the others?*

Which topics would you prefer to discuss?*

- Which topics would you rather not discuss?

At what time would you prefer to first discuss decisions about your future care?*

Who should initiate the conversation about decisions regarding your future care?*

How often and with what frequency would you prefer to discuss decisions about your future care with a healthcare professional?*
